# The Relationship of Fatty Liver with Body Composition Indices in Individuals with Metabolic Disorders - A Cross-Sectional Study

**DOI:** 10.12669/pjms.41.1.9261

**Published:** 2025-01

**Authors:** Nazlı Hacıagaoglu, Can Oner, Huseyin Cetin, Engin Ersin Simsek

**Affiliations:** 1 Nazlı Hacıagaoglu, MD Family Medicine, University of Health Sciences, Kartal Dr. Lütfi Kirdar City Hospital, Istanbul, Turkey; 2 Can Oner Associate Professor Family Medicine, University of Health Sciences, Kartal Dr. Lütfi Kirdar City Hospital, Istanbul, Turkey; 3 Huseyin Cetin Assistant Professor Family Medicine, University of Health Sciences, Kartal Dr. Lütfi Kirdar City Hospital, Istanbul, Turkey; 4 Engin Ersin Simsek Associate Professor Family Medicine, University of Health Sciences, Kartal Dr. Lütfi Kirdar City Hospital, Istanbul, Turkey

**Keywords:** Anthropometry, ABSI, BRI, MAFLD, Steatosis

## Abstract

**Objective:**

In this study, it was aimed to screen fatty liver in individuals with metabolic disorders, and to investigate the use of some anthropometric calculations and body composition indices in demonstrating fatty liver disease.

**Methods:**

The cross-sectional study included 224 participants with metabolic diseases. Anthropometric measurements of the participants were measured. Waist/Height ratio, Rohrer Index, Broca-Katsura Index, Relative Fat Mass (RFM), Body Roundness Index (BRI), A Body Shape Index (ABSI) were calculated. The presence of qualitative steatosis was evaluated abdominal ultrasonography. As a result of all analyzes, p <0.05 was accepted as significant.

**Results:**

The mean age of the 224 participants included in the study was 55.1±10.6 (min=23, max=83) years. 84 (37.5%) of the participants were female and 140 (62.5%) were male. Steatosis was detected on ultrasonography in 142 (63.4%) of the participants. Waist/Height ratio, Rohrer index, Broca-Katsura index, RFM, BRI, BMI and waist circumference were found to be significantly higher in participants with steatosis (p<0.05). According to the ROC analysis, BRI cut-off value of 4.6 were found to be significant in detecting steatosis.

**Conclusions:**

As a result of our study, we found that various body composition indices, especially BRI, were associated with fatty liver. Especially in female, it was seen that screening of fatty liver with cut-off values of body composition indices, and confirmation by ultrasonography, if necessary, may enable early diagnosis. In male, it was seen that body composition indices and fatty liver could not be determined directly, only BMI and waist circumference were indicative.

## INTRODUCTION

Over the past few years, the number of overweight and obese adults worldwide has surged to a staggering two billion. While excess weight is not classified as a disease per se, it significantly raises the likelihood of developing various illnesses, particularly insulin resistance.^1^ Over the last three decades, the rate of obesity and related metabolic disorders has risen sharply. The clinical symptoms of chronic energy surplus are insulin resistance, dyslipidemia, and obesity. Metabolic organs such as adipose tissue, pancreas, and liver are responsible for regulating chronic energy excess. However, when these organs’ capacities are exceeded, it can lead to the onset of dyslipidemia, type two diabetes, and metabolic-associated fatty liver disease.^2^ Metabolic-associated fatty liver disease (MAFLD) is a new terminology for non-alcoholic fatty liver disease and is associated with metabolic dysfunction.^3^

The increase in diabetes and obesity, which were part of metabolic features, are responsible for the increase in MAFLD.^4^ Unbalanced and unhealthy diet and intake of more calories than energy expended lead to the accumulation of triglycerides in adipose tissues and the liver. Studies have revealed that visceral fat accumulation may be high even in individuals with low body mass index (BMI).^5^ Large waist circumference and visceral adiposity significantly increase the risk of insulin resistance and MAFLD compared to BMI.^6^,^7^ Research shows that lifestyle and nutritional habit changes play a very important role in the management of MAFLD.^4^ Diabetes, insulin tolerance and resistance, β cell function, and fatty liver disease are the leading metabolic disorders. Metabolic disorders and related conditions should be screened with appropriate methods in the adult population.^1^

Abdominal ultrasonography is generally sufficient to detect hepatic steatosis. Large epidemiologic studies show that indices or scores that can be calculated with serum biomarkers can be used to detect steatosis when imaging methods are unavailable or cannot be used.^3^ Although waist circumference and visceral adipose tissue are valuable in predicting MAFLD, it is considered that more accurate indicators should be developed.^7^ Various body composition indices, including Body Roundness Index (BRI), calculated by anthropometric measurements such as waist circumference, BMI, and height, can be used in individuals with metabolic disorders including A Body Shape Index (ABSI). Various studies have shown that these indices are essential in detecting health problems such as diabetes mellitus, hyperuricemia, and cardiovascular disease. There are very limited studies on the power of body composition indices to predict MAFLD.^8^

This study aimed to screen for fatty liver disease in individuals with metabolic disorders and to investigate the use of some anthropometric calculations and body composition indices to demonstrate fatty liver disease.

## METHODS

This cross-sectional study included 224 participants aged 18 years and older with metabolic diseases associated with fatty liver disease, hypertension, diabetes mellitus, prediabetes, hyperlipidemia, and obesity, who randomly applied to polyclinic. Participants’ sociodemographic information and known chronic diseases were questioned. The presence of Metabolic Syndrome was determined based on NCEP-ATP-III criteria.^9^ The presence of qualitative steatosis was evaluated by Mindray DP-8500 2D abdominal ultrasonography. MAFLD was accepted if fatty liver disease is demonstrated in individuals who are overweight and diagnosed with Type two DM or who are of normal weight but have at least two metabolic diseases.^7^

The height, weight, and waist circumference of the participants were measured by the investigator. After the height and weight measurements of the patients, BMI was calculated with the formula body weight (kg)/square meter of height (m^2^). Waist/height ratio (WHtR) was calculated with waist circumference(cm)/height(cm) formula. Rohrer Index was calculated with weight(kg)/height^3^(m) formula. Broca-Katsura Index was calculated with weight(kg)/((((height(cm)-100))*0.9)-1)*100 formula. Relative Fat Mass (RFM) was calculated with 64–20*(height(cm)/waist circumference(cm)) for men, 76-20*(height(cm)/waist circumference(cm)) for women. ABSI was calculated with waist circumference(cm)/(BMI^2/3^*height^1/2^) formula. BRI was calculated with 364.2-365.5*(1-(waist circumference (cm)/2Л)^2^/(0.5*height^2^)^1/2^ formula. Written informed consent was obtained from all participants. Participants with known liver disease were not included in the study.

The study data were analyzed in SPSS 21.0 package program. Descriptive measures (frequency, percentage, mean, standard deviation, and minimum-maximum values) were used in the analysis. Kolmogorov smirnov was applied as normality test. Student’s t-test was used for normally distributed data. ROC analysis, specificity, sensitivity, area under the curve and 95% confidence interval (CI) were used to analyze the relationship between different anthropometric indicators and steatosis. As a result of all analyses, a p-value <0.05 was accepted as significant.

### Ethical Approval:

Ethics committee approval was obtained from the Ethics Committee of Kartal Dr.Lütfi Kırdar City Hospital (Decision No: 2023/5l4/248/2). The study was conducted following the Declaration of Helsinki

## RESULTS

The mean age of the 224 participants included in the study was 55.1±10.6 years (min=23, max=83). 84 (37.5%) participants were female, and 140 (62.5%) were male. Metabolic syndrome was found in 162 (72.3%), hyperlipidemia in 176 (78.6%), hypertension in 108 (48.2%), diabetes mellitus in 150 (67.0%), prediabetes in 45 (20.1%), obesity in 121 (54.0%) and MAFLD in 142 (63.4%). It was observed that 55 (24.6%) participants were smokers, and 11 (4.9%) were regular alcohol users.

When the anthropometric measurements of the participants were analyzed, the mean height was 164.0±9.7 cm, mean weight was 84.5±15.3 kg, mean waist circumference was 99.6±11.9 cm, and mean BMI was 31.5±5.3 kg/m^2^. The mean WHtR of the participants was 0.6±0.1, mean Rohrer index was 19.3±3.7, mean Broca-Katsura index was 151.0±27.2, and mean ABSI was 0.08±0.05**.** The mean RFM of females participants was 41.9±4.4, and the mean RFM of male participants was 31.0±3.9 (p=0.000). The mean BRI of the participants was 5.7±1.8.

Steatosis was detected on ultrasonography in 142 (63.4%) of the participants. 82 (36.6%) participants had normal parenchymal echogenicity on ultrasonography, 62 (27.7%) had Grade one steatosis, 66 (29.5%) had Grade two steatosis, and 14 (6.3%) had Grade three steatosis.

**Fig.1:**
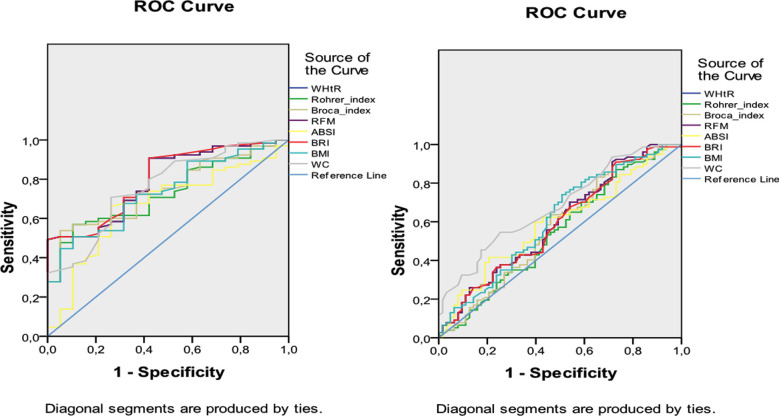
ROC curve of different variables in detecting steatosis in female and male.

WHtR, Broca-Katsura index, RFM, BRI, BMI, and waist circumference were significantly higher in participants with steatosis (p=0.001, p=0.019, p=0.000, p=0.001, p=0.001, p=0.000, respectively). The cut-off points, sensitivities, specificities and areas under the curve of different variables in detecting steatosis in male and female were shown in Table-I. ROC curve of different variables in detecting steatosis in female and male were shown in Fig.1.

**Table-I:** Cut-off point, sensitivities, specificities and area under the curve of different variables in detecting steatosis in male and female.

	Cut-off	Sensitivity (%)	Specificity (%)	AUC (95% CI)
***Male***				
WHtR	NS	NS	NS	NS
Rohrer Index	NS	NS	NS	NS
Broca-Katsura Index	NS	NS	NS	NS
RFM	NS	NS	NS	NS
ABSI	NS	NS	NS	NS
BRI	NS	NS	NS	NS
BMI (kg/m^2^)	30.8	64.9	54.0	0.610 (0.516-0.705)
Waist Circumference (cm)	97.5	64.9	54.0	0.683 (0.596-0.770)
***Female***				
WHtR	0.55	73.8	63.2	0.794 (0.687-0.900)
Rohrer Index	16.54	70.8	57.9	0.730 (0.616-0.843)
Broca-Katsura Index	130.87	72.3	63.2	0.743 (0.631-0.855)
RFM	39.95	73.8	63.2	0.794 (0.687-0.900)
ABSI	0.07	67.7	68.4	0.666 (0.531-0.802)
BRI	4.6	70.8	68.4	0.800 (0.694-0.905)
BMI (kg/m^2^)	28.0	72.3	63.2	0.734 (0.618-0.851)
Waist Circumference (cm)	94.5	70.8	73.7	0.760 (0.640-0.880)

WHtR: Waist/height ratio; RFM: Relative Fat Mass; ABSI: A Body Shape Index; BRI: Body Roundness Index; BMI: Body mass index; AUC: area under the curve; 95% CI: 95% confidence interval; NS: not significant.

## DISCUSSION

Non-alcoholic fatty liver disease (NAFLD) is recognized as an important risk factor for peripheral vascular disease, diabetes, renal disease, and cardiocerebrovascular disease, and its most important feature is hepatic steatosis. As a result of our study, WHtR, Broca-Katsura index, RFM, BRI, BMI, and waist circumference were significantly higher in participants with steatosis. It has been seen that body composition indices can be used to screen fatty liver with the determined cut-off values in female, while body composition indices and fatty liver cannot be directly determined in male, only BMI and waist circumference are indicative.

BRI is an index that can be used to define obesity and can show abdominal accumulation of adipose tissue.^10^ Various studies have been conducted on the effect of BRI on metabolic syndrome.^11^,^12^ Liu B. et al. found the optimal cut-off values for BRI for determination of metabolic syndrome 3.85 in male (sensitivity 76.5%, specificity 82.1%) and 4.05 in female (sensitivity 76.4%, specificity 70.3%).^11^ Suliga E. et al. have found 4.82 for male and 5.05 for female cut-off values for BRI. Similar to our study, it was observed that BRI was more valuable in female.^12^ Studies examining the relationship between Type two DM and BRI have also been conducted in recent years. In their study, Zhao W. et al. found a positive linear relationship between BRI and Type two DM.^13^ Chang Y. et al. have also shown that BRI is important in predicting the risk of Type two DM in both sexes, as is waist circumference and BMI.^14^

There are various studies on WHtR as a marker in screening for MAFLD, which contributes to the development of diseases such as Type two DM and metabolic syndrome.^8^,^10^,^15^ In their study, Sheng G. et al. investigated the role of various indices related to obesity and lipids in determining the risk of NAFLD. As a result of the study, they found that the Triglyceride-Glucose index best indicated the risk of NAFLD.^16^ Motamed N. et al. found that ABSI, BRI, WHtR, and Waist/Hip ratio were associated with NAFLD, and especially BRI and WHtR showed a higher association with NAFLD. In their study, they found the optimal cut-off points for BRI to be 4.0 in male and 5.0 in female, and the optimal cut-off points for WHtR as 0.533 in male and 0.580 in female.^8^ Li H. et al. found that the potency of BRI and WHtR in predicting MAFLD was higher, especially in male.^17^ Xie F et al. found a strong association of BRI with NAFLD, though the association with BMI was stronger. Regardless of gender, the optimal cut-off value for BRI was 3.67, the optimal cut-off value for BMI was 24.9, and the optimal cut-off value for ABSI was 0.0799, and results similar to our study were found.^18^ Lin I et al. found a relationship between metabolic syndrome and NAFLD and high BMI, WHtR, BRI, and Triglyceride index.^19^ Chang Y et al. found that ABSI was not superior to waist circumference and waist/height ratio in determining the risk of Type two DM, and the predictive power of ABSI was weak.^14^ Zhang Y et al., in their study investigating the place of different anthropometric measurements in NAFLD screening in elderly individuals, found significant BMI cut-off value of 23.99, waist circumference cut-off value of 87.75, WHtR cut-off value of 0.57, and RFM cut-off value of 40.89.^20^ Kuang M et al., on the other hand, showed that BMI and waist circumference in female and BMI and ABSI in male had better diagnostic performance in demonstrating NAFLD. The use of BMI together with ABSI was found to be more valuable in the general population.^21^ It is thought that different anthropometric measurements can be used in the evaluation of NAFLD risk, as well as the use of combined methods can be guiding. Different results have been found in different studies. More studies are needed to show the relationship between body composition indices and fatty liver.

## CONCLUSION

We found that various body composition indices, particularly BRI, are associated with fatty liver disease. Early recognition and prevention of problems that may develop in individuals with metabolic disorders are important in reducing mortality and morbidity. Evaluating fatty liver disease in primary health care with simple, easily applicable body composition indices is non-invasive and allows early recognition of steatosis. Their use in female is more informational, and they do not replace ultrasonography in male.

### Authors’ Contribution:

**NH, CO, HC, EES:** Substantial contributions to conception and design, or acquisition of data, or analysis and interpretation of data; drafting the article or revising it critically for important intellectual content; and final approval of the version to be published. All auithors have read the final manuscript and are responsible for the integrity of the study.
